# Unveiling polish judges’ views on empathy and impartiality

**DOI:** 10.3389/fsoc.2024.1417762

**Published:** 2024-11-01

**Authors:** Mateusz Stępień

**Affiliations:** Jagiellonian University, Kraków, Poland

**Keywords:** empathy, judging, judges, decision making, in-depth interview

## Abstract

The exploration of empathy’s significance in judicial decision-making has garnered attention in scholarly discourse, yet there is a noticeable gap in studies delving into judges’ perceptions of empathy’s role, advantages, and impediments. This neglect reflects an “anti-empathetic” discourse that overlooks the insights of those central to justice delivery. Consequently, there is an urgent need for empirical inquiries into judges’ perspectives on empathy, its definition, and its integration into their work. Primarily concentrated in Anglo-Saxon jurisdictions, empathy research in judicial decision-making lacks diversity. This paper responds to two critical calls: understanding judges’ views on empathy and expanding research beyond common-law systems. It presents empirical research investigating Polish judges’ perspectives on empathy, with a focus on its relationship with impartiality. This inquiry is crucial given debates on whether empathy compromises impartiality, particularly evident in discussions surrounding judicial appointments. Based on in-depth interviews with Polish judges, this article identifies five strategies employed by judges to reconcile empathy with impartiality, termed as “paths”: (1) claiming symmetry in distributing empathy between parties, (2) defining empathy as unemotional, (3) mitigating empathy’s influence on judgments, (4) emphasizing control over empathy, and (5) deabsolutizing formal impartiality and making more room for empathy. The paper discusses these strategies and comments on them, shedding light on the nuanced ways in which judges navigate the intersection of empathy and impartiality in their decision-making processes.

## 1 Introduction

The examination of empathy’s role in broadly understood judicial decision-making has garnered significant interest in the literature (e.g., Henderson, 1987; Bandes, 2009, 2011; Booth, 2019; Stępień, 2021). However, only an exceptionally limited number of studies focused on providing access to how judges perceive the role, advantages, or obstacles of empathy in their work (Bergman Blix, 2019; Roach Anleu and Mack, 2021: Chapter 3). This marginalization implies that the almost entire discourse on empathy in judging overlooks the views and experiences of individuals whose role in delivering justice is crucial. Therefore, there is a pressing need for empirical research oriented toward understanding what judges think and experience concerning empathy, including what this term encompasses for them and how they perceive and situate empathy within their work.

Moreover, it is crucial to highlight that the majority of studies focusing on empathy in judicial decision-making, especially empirical ones, are primarily centered around the Anglo-Saxon sphere. A notable exception comes from Sweden (e.g., Wettergren and Bergman Blix, 2016; Bergman Blix and Wettergren, 2018: 11–12, 107–109, 119, 144, 166). There is a critical need to broaden the discussion and empirical research on judicial empathy beyond common-law jurisdictions. This expansion would bring forth a new set of judges’ experiences and thoughts on empathy embedded in different organizational and institutional settings. Such diversity in research efforts is vital for achieving a more comprehensive and less confined understanding of the role empathy plays in the context of judicial decision-making.

In response to the two aforementioned calls—the need for investigating judges’ perspectives on the role of empathy in their work and broadening the scope of interest beyond common-law jurisdictions—the research underpinning this paper aimed to empirically investigate the views and experiences of Polish judges of common courts (which administer justice within the scope beyond the authority of administrative courts, military courts, and the Supreme Court) regarding empathy in judicial decision-making. Specifically, this paper zooms in on judges’ views on the relationship between empathy and impartiality. Focusing on this topic is justified because a huge part of the discussions in the literature is oriented toward the claims that empathy and impartiality are “foes” (as the former brings biases, unequal treatment of parties, or emotional impact which corrupts impartiality) or they remain “friends” as some argue (Franks, 2011; Lee, 2013; Colby, 2012; Maibom, 2022: Chapter 10). Importantly, the claim that judicial empathy undermines impartiality was expressed loudly during the public debates in the United States around Barack Obama’s statement that the possession of empathy should play a role in the appointment of judges [see reconstruction of these critiques on empathy in: West (2011) and Fissell (2017); see also Zipursky (2012)]. The paper places judges’ perspectives at the forefront to facilitate a comprehensive exploration of this pressing issue.

The paper begins by offering sketch of the main points of the discussion on empathy in judicial setting. It then delves into the Polish judicial system and some basics about Polish judges. Subsequently, it provides a broad outline of the research methodology employed to examine judges’ perspectives and experiences regarding empathy in their professional practice. Once these introductory themes are established, the paper delves into judges’ comprehension of empathy and impartiality before proceeding to reconstruct their perception of the relationship between these two. The research identifies five strategies employed by judges to reconcile empathy with impartiality: (1) claiming symmetry in distributing empathy between parties, (2) defining empathy as unemotional, (3) mitigating empathy’s influence on judgments, (4) emphasizing control over empathy, and (5) deabsolutizing formal impartiality and making more room for empathy. The paper examines these strategies and provides commentary, shedding light on the nuanced ways judges articulate their approach to balancing empathy and impartiality in their decision-making processes. The final section outlines potential reasons behind the key findings and highlights the main challenges associated with the strategies discussed. Overall, this investigation aims to address a neglected yet crucial aspect, essential for a comprehensive understanding of the current and desired role of empathy in judges’ work from multiple perspectives.

## 2 Studies on empathy in judicial setting

There is no place here to fully comprehend the literature on law and empathy. Even presenting writings on empathy in a judicial setting can be challenging due to the vastness of these studies and the multitude of explored threads. In addition, comprehending these conversations necessitates a certain level of proficiency in the overall field of empathy studies. It is not the purpose here to provide a comprehensive overview of empathy literature or research in legal settings, but some general comments are required to clarify the viewpoint proposed in this paper.

Firstly, the term “empathy” made its entry into the English lexicon in the early 20th century, primarily as a translation of the German term *Einfühlung* (Lanzoni, 2018). What might not be immediately intuitive is that the original context of *Einfühlung* resided in the realm of art and aesthetic experiences. It wasn’t until later that the word found its way into the vocabularies of a plethora of fields. However, if we associate the meaning of empathy with “putting oneself in another’s shoes,” a process through which one person comprehends another’s situation or perspective, or alternatively “emotional attuning with others,” it becomes evident that these have been a part of human beings’ experiences long before the coining of the term. When we examine the history of philosophical thought, for instance, David Hume’s or Adam Smith’s notion of sympathy, the Confucian concept of *shu*, or even the Golden Rule, they all cover similar processes to what we now associate with empathy.

Secondly, considering the approximation of empathy, it seems to be beneficial for judges and judicial decision-making. To put it briefly, taking another person’s perspective, in certain conditions, can lead to a better understanding of the case. Moreover, transcending a decision based on one’s view by entering the perspectives of others can be seen as crucial for impartiality. In addition, sensitive management of hearings calls for recognizing the emotions of all participants, which can be achieved through empathy. In these lines, some authors argue that judges’ empathic responses are crucial for implementing procedural justice. According to Megan Pearson (2020), empathy plays a critical role in achieving several elements of procedural justice, particularly about treating litigants with respect, fostering trust among the parties involved, and enabling open expression. Nevertheless, Pearson also emphasizes that while empathy offers these advantages, it should not be confused with emotion itself. In discussions of procedural justice, the active and empathic listening of judges is often emphasized as a necessary quality to ensure fair proceedings.

However, on the other hand, empathy is viewed as an undesirable quality of judges, with its role in the decision-making process painted in dark colors. In general, perhaps the initial reaction of many lawyers is that “empathy” is a concept that initially seems counterintuitive in the legal world (Henderson, 1987: 1576). Using empathy can be seen as introducing distortions into the legal process when employed by legal decision-makers. From this perspective, its influences should be tamed rather than elaborated upon, as it brings unbridled subjectivism into judging and opens decision-making processes to well-known empathy biases. These biases include the similarity bias, which means that one tends to empathize more with a person who shares similar characteristics, and the here-and-now bias, which covers situations in which one empathizes more with people who are present and less with those who are absent. From this viewpoint, the influence of empathy on legal decision-making threatens judicial impartiality, objectivity, and neutrality. In this reading, empathy in the judicial context is considered “a wild, untamed, destabilizing force that cannot coexist with the rule of law” (Bandes, 2011: 105). Indeed, these claims made within the judicial context reflect broader discussions and critical voices [see Bloom and Prinz (n.d.)] regarding the role of empathy and its centrality in fundamental conversations about morality and core elements of human cooperation.

Thirdly, it is essential to underscore that the role of empathy in judicial decision-making relies on how it is perceived and ramified. The literature suggests that there is a range of understandings of empathy in research on empathy’s impact on judicial decision-making. Scholars, especially those with a social sciences and psychology orientation, tend to prioritize empathy as an automatic emotional response characterized by emotional sharing or mirroring, highlighting the potential negative impact of empathy on judicial decision-making. The main reason for this is that (affective) empathy introduces biases (like familiarity bias, here-and-now bias) into decision-making, contradicting the fundamental principles and values of the judiciary. For example, studies of mock jurors demonstrate that inducing empathy toward a defendant can influence the jury’s decision (Wood et al., 2014; see also Glynn and Sen, 2015). In this perspective, empathy is seen as an automatic response leading to identification with the defendant based on certain stimuli. In contrast, a vast tradition views empathy in the context of judging differently. The famous mentioned above declaration by Barack Obama that the selection of judges should prefer individuals who possess empathy, that is, who understand the broader social context and who possess diverse life experiences (Rollert, 2014). Certainly, this implies a different reading of empathy, as here it is seen as a disposition to delve deeper and consider the larger social and historical context in judging. In this view, empathy involves an intellectual process that is navigated and based on deliberate choices. It is evident that the former example links empathy with emotional reactions that are hard to control, while the latter suggests a more intellectual process (but not necessarily devoid of emotions) guided by deliberate choices.

The examples make it clear that a grasp of what empathy entails is essential for considering its impact on judicial decision-making. Some view empathy as an asset in the judicial system, while others perceive it as a weakness depending on their interpretation of empathy. Given these circumstances, it is even more critical to reconstruct judges’ perceptions of empathy and how they tie it to impartiality.

## 3 Polish judiciary—a glimpse

To comprehensively understand even a fragment of the views, perspectives, and experiences of Polish judges of the common courts regarding the role of empathy in their work, as well as to comprehend the methodological choices, it is imperative to possess basic knowledge about both the structure and characteristics of the Polish judicial system, the place of common courts within it, and the basic conditions of the profession of judges. This knowledge is essential for carefully integrating the collected findings into the growing body of research on empathy in judging and understanding how to approach this topic by empirical methods.

The Polish legal system follows the civil law tradition, and consequently, the Polish judiciary displays typical characteristics of civil law jurisdictions [see Merryman and Pérez-Perdomo (2018)]. In accordance with Article 10 of the Constitution of the Republic of Poland (issued 2 April 1997), the system of government in the Republic of Poland is founded on the separation and balance between the legislative, the executive, and the judicial powers. The judicial powers encompass courts and tribunals, represented by the Constitutional Tribunal, responsible for scrutinizing the constitutionality of laws. The courts include military courts, administrative courts, and, finally, the common courts and the Supreme Court. The Supreme Court oversees the ordinary courts, manages cassations of specific judgments rendered in the second instance, and, since 2018, handles the so-called extraordinary complaint, which can be applied to valid and final judgments. The jurisdiction of common courts is the most extensive, and typically, Polish citizens come into contact with them for matters such as divorces, adoptions, damages, violations of contracts, offenses, and crimes.

There are three following types of common courts. First, regional courts (*sądy rejonowe*) serve as the courts of first instance, with extensive original jurisdiction, handling most cases except those reserved for other specialized courts. Their jurisdiction typically covers an area encompassing several communes. These courts are organized into specialized divisions such as Intellectual Property Courts and the Competition and Consumer Protection Court. Regional courts, as the primary trial courts, are frequently involved in a wide array of cases with a multitude of participants, primarily witnesses. The establishment of the factual basis of the cases leads to a high density of human interactions in the courtroom, often accompanied by participants’ emotional displays. Regional courts process a large number of cases daily. The courtrooms in regional courts tend to be small and bear the marks of time. Hearings are characterized by a lower degree of contentious legal disputes and less legal wrangling compared to higher levels of the judiciary. The scope of the presence of ritual and symbolism is limited. Typically, with the exception of some labor and family cases (where lay judges employed for a period of time, and not *ad hoc*, are prescribed), cases are handled by a single judge sitting alone, engaging face-to-face with participants. Second, district courts (*sądy okręgowe*) function both as first- and second-instance courts, handling more serious cases as well as appeals of judgments from regional courts. Their jurisdiction covers an area of several district courts. Third, appellate courts (*sądy apelacyjne*) function as second-instance courts, and they do not possess original jurisdiction. Their appellate jurisdiction covers a territory of at least two district courts. In general, they do not examine the evidence and rely on the material gathered by lower courts. Appellate courts resemble “the courts basing mainly on papers.”

The court proceedings in Poland are governed by procedural codes, with the most important being the Criminal Procedure Act of 1997 and the Civil Procedure Act of 1964 (see Bednarek, 2014; Ryan, 2016). There are two types of civil procedures in Poland: contentious proceedings and non-contentious proceedings. In general, the former is adversarial in nature, while the latter, due to not involving two opposing and conflicting parties, is less adversarial. The criminal procedure is mixed, possessing both inquisitorial and adversarial elements, as it is based on the two-party model, involving an accused and a public prosecutor. In cases of severe crimes, the professional judges are accompanied by two or three lay judges who are selected for a period of 4 years.

In Poland, trial judges assume an active, if not hyper-active, role in both civil and criminal cases. Polish trials are characterized by the extensive involvement of the judge (or judges when sitting in a panel) in establishing the factual basis of cases. Judges often take the lead in proceedings, including the evidentiary phase, where they actively question witnesses, among other tasks [further details and examples on this matter can be found in: Dudek and Stępień (2021)]. This stands in contrast to the roles of judges in common-law systems, where their primary function is to ensure adherence to procedural rules.

Returning to the process of becoming a judge, it is crucial to understand that the selection and advancement of judges in the Polish judiciary resemble the civil service [see Mistygacz (2020)]. Individuals aspiring to become judges undergo extensive specialized training, typically lasting a minimum of 10 years, which includes obtaining a master’s degree in law. Law schools in Poland have a 5-year duration, and a law degree serves as the initial step toward a career in the legal professions. After earning a law degree, aspiring judges must successfully pass a highly competitive entrance exam to gain admission to the state-managed National School of Judiciary and Public Prosecution, established in 2009. Previously, specialized training for those who passed the entrance exam was conducted in appellate courts. Since 2009, a centralized, 4-year school-like education has been established. Following rigorous training and a final examination, successful candidates can apply for open positions in the judiciary. They serve as judicial assessors before being appointed to full judge positions. There is also the possibility of being appointed as a judge after practicing other legal professions for some period. In both cases, candidates for open positions in the judiciary are assessed and recommended by the National Council of the Judiciary of Poland. Only after receiving a positive evaluation, they get nominated by the President of the Republic of Poland for an unspecified period of time. The constitutional status of the currently functioning National Council of the Judiciary, based on the legal act issued in 2018, has been a source of division among lawyers, politicians, and the wider public, leading also to evaluations by European courts (ECHR, ECJ). Nevertheless, judges undergo periodic evaluations of their work and decisions throughout their careers, with promotions being determined by the National Council of the Judiciary of Poland. The career of a judge typically begins at the lowest level of the judiciary, such as regional courts.

Significantly, the positivist tradition holds considerable influence in Poland regarding how judges are trained, how they perceive their role, and manage hearings. This influence has deep historical roots, as legal professionals used a “formalistic shield” during the communist era (1945–1989) to safeguard their professionalism. The substantial impact of German legal traditions before World War II further contributes to this influence. The convergence of these factors shapes the role of judges and their judicial conduct, emphasizing the long-standing dominance of the “stone-face” (or “poker face”) ideal, which underscores the importance of showing dispassion, employing highly formal communication, and maintaining a high power distance between lawyers and laymen [see Dudek and Stępień (2021)].

## 4 Basics about the conducted research

The research commenced by prioritizing the views and experiences of judges regarding the role of empathy in their work. The subsequent step involved selecting an appropriate research technique aimed at gaining insight into their internal world. Given the nuanced nature of the subject matter, qualitative research was deemed most suitable for comprehending perceptions, opinions, sentiments, and experiences of judges. The study employs in-depth interviews as primary source of data [for more general insights on interviewing legal professionals and legal elites, see Korkea-aho and Leino (2019), Kenney (2020), and Gupta and Harvey (2022)]. Judges were invited to participate in semi-structured dialogs following a predetermined set of questions that delved into various dimensions of empathy and its relationship to judicial decision-making. Initially, the study leveraged the snowball method to tap into existing contacts within the judiciary to identify potential interviewees for recruiting judges. Subsequently, after randomly selecting courts from a pre-established pool, general invitations were sent to judges from these selected courts to participate in the research.

The interview began by inquiring judges about general topics related to empathy and its connections to other processes. Questions about fundamental intuitions, beliefs, and even instances from their personal lives regarding empathy were posed to encourage a diverse range of interpretations in this domain. This approach aimed to dissuade judges from immediately framing their responses within the judicial context and its associated professional culture, with the goal of avoiding the activation of dominant cognitive patterns and instead eliciting language and concepts less central to their professional roles. In the subsequent phase of the interviews, judges were prompted to share their experiences and reflections concerning judicial behavior directly or indirectly associated with empathy within a judicial context. This included discussions on how they address human suffering, anguish, and challenges typically observed during trials, as well as whether and how they respond to courtroom events. They were also asked about their experiences of “putting themselves into someone else’s shoes” and whether they show a “human face” during hearings. Only after exploring these topics were judges asked for their opinions on whether judges should exhibit empathy. Finally, the issue of the relationship between empathy and impartiality in the judicial context, along with inquiries about their training and needs in this regard, served as the focal point of the interviews. The objective was to gather examples of specific judicial behaviors, cases, and real courtroom situations, as well as to glean insights into the personal experiences of judges.

Due to the impact of the COVID-19 pandemic, the research protocol shifted from the planned face-to-face interviews to internet-based in-depth interviews [see Salmons (2016) and Howlett (2022)]. This adaptation allowed for flexibility and comfort for the participants, as most interviews were conducted from their homes after work hours. Surprisingly, this change to a virtual setting created a relaxed atmosphere, fostering openness among the participants. Interviews took place between mid-2021 and early 2022, with a total of more than 40 conducted, but only 37 were included in the data analysis because of technical problems with recordings and internet connection.

The interviewed judges represented diverse specializations, including civil (14), family (9), criminal (8), commercial (5), and labor (1), with all but three working in regional courts. The participants had an average work experience as a judge of almost 16 years. The length of the interviews varied depending on factors such as time constraints of participants and the depth of the narratives provided by them, with an average duration of approximately 100 min and some extending up to two and a half hours. While the interview followed a structured format, interviewees were urged to freely share their perspectives, with the researcher actively engaging in discussions and seeking elaboration. All interviewees consented to recording the interviews, enhancing accuracy in reporting the results. The data collected from the interviews were transcribed and anonymized.

Thematic analysis was chosen and employed as the main method for organizing, analyzing, and interpreting the data at the research stage due to its potential to “identify, analyze, and report patterns (themes) within the data” (Braun and Clarke, 2006: 79). The aim was to identify and map judges’ views on empathy-related subjects and to extract key themes—meanings that capture the core ideas within the data in relation to the research question and represent a patterned response or cluster within the dataset. Thematic analysis is commonly used to condense extensive and varied raw data into an ordered, structured format. In this study, after the initial familiarization with the transcripts, themes related to the research questions were identified. These themes often enabled the creation of typologies of the interviewees’ views. Subsequently, the transcripts were re-read, and the list of themes (and typologies) was refined and reinterpreted.

It is important to stress that the judges who decided to participate in these in-depth interviews and invest their precious time in this manner can be seen as not typical representatives of the judiciary. This suggests that it should be considered whether the interviewed group was in some sense exceptional, consisting of judges who, hypothetically, are more sensitive, curious, and perhaps empathic. This hypothesis is unsupported and relies on the assumption that participation in this study indicates a willingness to engage in activities that may not have clear instrumental benefits, while also recognizing the potential for empathy within the judicial system.

Moreover, the study was conducted under extraordinary conditions, particularly considering the sociopolitical backdrop in which the research team operated while examining judges’ views on empathy in their work [see Sadurski (2019) and Zoll and Wortham (2019)]. Over the past few years, judges have found themselves embroiled in tense political and legal disputes. While specifics are challenging to provide, foundational information is necessary to contextualize the study.

The issue began in the autumn of 2015 when the ruling majority, led by the right-wing *Prawo i Sprawiedliwość* (Law and Justice) party, initiated significant legal reforms primarily aimed at the judiciary. The first major battleground was the Constitutional Tribunal, vested with significant powers, including the authority to review the constitutionality of laws. Subsequently, the attention of the ruling party shifted toward judges of the common courts, who were often portrayed in government narratives as an “unaccountable” occupational group, commonly referred to as a “caste” [for a comparative insight into attacks on judges in Central Europe, see Čuroš (2023)]. By the summer of 2017, the government introduced legislative proposals regarding the recruitment and appointment of judges, the organization of common courts, and a comprehensive reorganization of the Supreme Court. The legislative process was marked by an aggressive propaganda campaign by the government against judges, which emphasized issues such as judicial errors and alleged misconduct. Despite unprecedented public protests and the mobilization of judges (Matthes, 2022; Puleo and Coman, 2024), all three significant reforms affecting the judiciary came into effect by the beginning of January 2018, following vetoes of two out of three bills by the President and proposing slightly different details. These reforms further exacerbated the crisis surrounding the judicial branch and introduced new areas of conflict. In general, the measures introduced through these legislative changes were perceived as attempts to exert even greater political control over the judiciary and undermine its independence [see Szwed (2023)]. The change in the ruling majority in late 2023 did not bring about the resolution of the issues, largely because the man holding the presidential office is aligned with the Law and Justice party. Moreover, the task of restoring the rule of law becomes a source of new controversies.

The impact of the political-social context on the research remains uncertain. It is hard to determine whether this affected the willingness of judges to participate and resulted in presenting a more positive view of judges who were under attack. During the interviews, judges primarily focused on their actual experiences and views related to empathy and judging, often refraining from delving into political discussions. However, they sometimes acknowledged this tense atmosphere without providing details or expressing complaints.

## 5 Judges’ understanding of empathy

The analysis uncovers the various ways judges understand empathy that can be categorized into several themes. These classifications offer insights into the diverse perspectives, choices, and tensions within judges’ views on empathy.

The first theme is close to perspective-taking. Some judges define empathy as the ability to understand others by putting oneself in their shoes, seeing things from their perspective, and attempting to comprehend their feelings and life situations. For instance, one judge [2] understood empathy as “an ability to put oneself in the place of the other person, seeing things from their eyes; an attempt to understand what they can feel in a given moment, how they can perceive a given situation.” Similarly, another judge [17] described empathy as “an attempt to understand other humans in the sense of putting oneself into their position and their life situation.” A family judge [15] summarized empathy as “embodying someone else’s way of thinking, delving into their emotional states and reasoning,” equating empathy with the “ability to look at reality through someone else’s eyes.”

Next, judges often describe empathy as “*wczucie się*,” a Polish term implying emotional connection and understanding someone else’s feelings deeply (entering emotionally into). This perspective goes beyond mere emotional identification and involves comprehending the needs and situations of others. For example, one judge [33] delved into the “feeling into” approach by describing empathy as “an ability to feel into the needs and situation of another person as well as the being, that is an animal.” Another judge [3] characterized empathy as “understanding the needs and feelings of the other side … looking at the other human not through the prism of one’s own ego, but to understand and feel into the situation of the human on the other side.” One judge [12] proposed a two-element view on empathy as “a skill, ability to reading the others’ emotions, emotional states and the skill of feeling into them, understanding them.”

The third way to grasp empathy is correlated with the Polish term “*współodczuwanie*,” which suggests feeling or experiencing emotions in unison with others (feeling together). This emphasizes the emotional aspect of empathy, with some judges focusing on understanding others’ emotions and sharing in their emotional experiences. For example, a judge [5] equated empathy with “co-feeling … with such an understanding of someone’s emotions … the ability to feel into these emotions.” Some judges initiated their descriptions of empathy by emphasizing co-feeling and then added the perspective-taking component. A judge equated empathy with “a skill to co-feel into others’ situations and understand their situation; looking a little at the case from their eyes.” Similarly, another judge [4] defined empathy as “co-feeling, that is feeling into the situation of another person, at least attempting to do so, as we frankly never can state and feel what the other person feels,” adding also “at least attempting to take into account their situation, their feelings.”

Next, some judges view empathy as being open-minded and understanding others’ situations without making quick judgments. This approach emphasizes understanding, listening, and being open to different perspectives. For instance, one judge [6] described empathy as “understanding what other humans feel or experience,” stressing the importance of delving into someone’s perspective, especially in situations where one “does not support [something], does not accept [something],” highlighting empathy as a reason-related and motivated skill. Similarly, another judge [7] characterized empathy as “understanding what other humans feel or experience,” emphasizing that it serves as a way to “avoid issuing fast, cheap, and superficial judgments.”

Last but not least, judges’ understandings of empathy indeed vary widely, with some presenting unique perspectives that diverge from the typical consensus found in professional literature. For instance, one judge [14] described empathy as “being open to other human beings, understanding what triggers them, discerning their needs, attempting to feel into their situation … comprehending the mechanisms that guide them … finding a path to agreement, and perhaps offering assistance—this entails seeing deeper, as well as not being easily offended.” Similarly, another judge [1] viewed empathy as a “sense of service for the other human … emerged from the respect for another human.” These perspectives highlight empathy as a comprehensive understanding and service toward others, which may not align precisely with conventional definitions.

In summary, the majority of the presented understandings align closely with tropes found in academic literature [see, e.g., Cuff et al. (2016) and Guthridge and Giummarra (2021)], particularly with the umbrella-like understanding of empathy that encompasses perspective-taking, sharing emotions, and emotionally tuning. However, some inconsistencies and over-inclusivity can be identified within these understandings. Notably, several approaches emphasize that empathy involves grasping the other’s situation or needs, which is more demanding than simply understanding their states of mind. Additionally, certain judges highlight empathy’s emotional aspect, which raises questions about its integration with impartiality in the judicial context. On the other hand, some judges emphasize empathy as a cognitive process, focusing on understanding others’ situations and perspectives without necessarily sharing their emotions. This understanding of empathy appears to align with judicial values such as impartiality and may even be seen as a means of ensuring it. Interestingly, a few judges introduce unique perspectives that highlight empathy’s role not only in understanding others but also in fostering collaboration and mutual respect, which is less controversial when applied to the judicial sphere. These viewpoints provide valuable insights into the judges’ diverse interpretations of empathy.

## 6 Judges’ understanding of impartiality

Remarkably, the interpretation of impartiality by judges has not yielded substantial insights in the literature, with few exceptions [such as Mack et al. (2021) and Roach Anleu and Mack (2021: 67–70)]. However, it is worth noting that although this subject was not the primary focus of the interviews conducted for this research, judges indeed presented varied approaches to impartiality, which can be organized into several typologies.

The first criterion considers the scope, scale, and depth of judges’ reflections on impartiality expressed during interviews. In this regard, presented views can be distinguished as (i) succinct, often also flat, reducing complex issues to simple “truths,” and (ii) complex, aiming to problematize the discussed issue, considering conditions and intervening factors, and highlighting problems. It can be observed that the approaches to impartiality presented by the judges were not as deep and nuanced as one might expect. Perhaps for judges, this is a non-controversial issue, and tacit knowledge dominates their thinking about it. Only a minority of judges attempted to shed some light on their understanding of impartiality, rather than treating it as a self-explanatory concept. For some, impartiality is equated with “non-favoritism” [37] or “seeing the interest of both sides” [30].

Next, typology refers to the fundamental difference between (i) impartiality related to reaching the final decision, and perhaps other elements of the court proceedings, but considered in terms of the actual processes occurring “inside” the decision-maker (“internal” impartiality) and impartiality as (ii) ensuring that the judge is not perceived by others as biased without delving into the actual reasons for the decision (“external” impartiality). In the latter case, emphasis is placed on how judicial behavior, decisions, words, and gestures are perceived by others (i.e., whether they are biased or whether they may seem so) [see Roach Anleu and Mack (2017: 9)].

The majority explore “external” impartiality. For example, one judge [13] expressed the view that “we [judges] should secure impartiality … this should be stressed and manifested at each step, that we are not on any side.” Another judge [29] emphasized: “I have to be very careful not to violate this principle of impartiality in [someone’s] perception because someone can perceive my actions as partial.” Referring to the use of empathy, this judge frankly points out: “I do not always see the need [for using it] and not always I have measures… But I must be very conscious and careful, in order not to violate the principle of impartiality in perception, in perception [of others], because my actions can be read out as partial.” An interesting perspective was expressed by another judge [15] who said: “an empathic judge can be impartial toward the parties, but if he would show this empathy too much, as I said, the other side of the trial can think that the judge favors this party, thus is not impartiality. Thus, we cannot show to some extent empathy, emotions, and we must keep a stone face.” The stone face, often attributed to the opposite of empathy, is used here as a strategy to ensure the hidden working of empathy, which does not corrupt the perception of the judge as impartial. In this case, there is no attempt to control empathy or manage empathic impulses, but not showing it is enough to guarantee that the participants would not make a legitimate accusation of partiality. The judge assumes that the problem lies only in expressing empathy, which by definition does not interfere with the decision-making process.

In the following typology, judges hold varying views on the concept of judicial impartiality—whether it is assumed, seen as a given, or viewed as a process that can be developed (and possibly diminished) similar to a form of work or performance. In the latter case, new topics and tensions arise, and the establishment of impartiality is seen as (not easy) work that judges must undertake. Often, the first approach relies on normative considerations that judges should be impartial, but obtaining this state is not treated as a kind of effort, work, or something worthy of deeper attention, as it just magically happens.

Referring to the understanding of impartiality as a certain process (performance), considering the criterion of what is possible in this regard in reality, two positions can be distinguished. According to the first, impartiality is a state relatively easy to achieve for the judge (e.g., just maintaining a “poker face”) or, as the second implies, it is an impossible state to fully achieve, even with the use of certain tools by judges and the presence of certain institutional conditions. The latter approach is realistic, not afraid to admit that full impartiality is not achievable, which, however, does not imply strong arbitrariness or the meaninglessness of attempts to achieve it.

There aren’t many adherences to the “easy” task thesis. However, one judge [26] expressed a similar view while discussing the human face, acknowledging that despite expressing a kind attitude toward the participants, “there is a need not to make room for the feelings of the parties that the side of one party is taken,” and what is important here—“this is not so hard [to do].” The majority stressed the hardship of being impartial. One judge [14] argued that absolute impartiality is a utopia as “everyone is shaped, has some opinions, ethical and moral, something for him is good and bad … talking about absolute impartiality … there is no chance.” Fortunately, some shared their deeper views. A judge [20] admitted that “everyone is subjective; it is not true that everyone is objective. And the judges are not an exception. However, [they] must tend to look as objective as possible, and not to consider some circumstance that could corrupt this objectivity.” This statement is tricky—we do not know whether the judge means that leaving aside subjectivity is impossible and only being perceived as objective is what can be done by judges. Another judge [29] mentioned: “I am always tending to be impartial, however, this is hard, because the heart often whispers something different” stressing the need for internal working on the “impulses of the heart.”

Summing this up, generally the judges’ view on the central category of judiciary—the impartiality—is not sophisticated and elaborate as one could imagine. Especially intriguing is that most of the judges focus solely on “external” impartiality, putting less effort into monitoring their internal processes.

## 7 How judges justify that empathy does not corrupt impartiality

The research revealed that judges do not view their empathy as conflicting with impartiality. It seems essential to contextualize this finding within each judge’s unique interpretation of empathy and assumptions regarding impartiality, as the understandings of empathy articulated at the outset of the interviews likely influence subsequent discussions on judicial context and impartiality. However, rather than delving into individual judges’ nuanced understandings of both concepts and their interrelationships, it is more productive to focus on the overarching tendencies and types of approaches judges employ to reconcile empathy and impartiality. This broader analysis can provide insights into prevailing attitudes within the judicial community regarding these fundamental aspects of judicial decision-making.

Five distinct ways in which judges attempt to explain or substantiate the absence of contradiction between empathy and impartiality can be distilled from the data. The presence of multiple “paths” in this regard does not imply that judges did not combine two or even three of them in their statements. On the contrary, they sometimes referred to several arguments simultaneously.

(1) According to the symmetry thesis, which is reflected in the literature (e.g., Lee, 2013: 163), impartiality is not compromised when a judge extends empathy not only to one party but to all participants involved. Under this thesis, the use of empathy by judges does not undermine a judge’s impartiality, especially in the “external” dimension, as long as it is applied uniformly to each party. In this framework, there exists a harmonious balance between empathy and impartiality, wherein each party receives the same or a similar depth of understanding and consideration. This perspective arises from concerns regarding investing empathy exclusively in one party, which could introduce bias into the decision-making process.

However, achieving this balance is not without challenges. As one judge [18] emphasized, that only if one is “empathic to both parties—then he can secure impartiality.” However, this is not an easy job, and “it can bring a negative consequence, thus [use empathy] with moderation.” Another interviewee [14] noted that genuine empathy extends to all parties involved, suggesting that managing empathic inclinations is necessary to ensure equal distribution. This view assumes that one must manage the empathic inclinations to achieve its equal distribution. The next judge [33], with diverse experience, also adhered to the symmetry thesis by suggesting that true impartiality arises when a judge behaves “empathically in the same way toward both parties.” This underscores the notion that emotions and personal worldviews must be set aside by judges to prevent bias, requiring a conscious effort to suppress inclinations that may arise due to emotional connections or shared values with one of the parties involved. Thus, some reactions need to be blocked in equal scope (negative aspect of symmetry thesis).

Importantly, some judges, even starting from a symmetry thesis, go deeper and subject it to fairly strong criticism. One judge [36] referred to these issues during the conversation. He starts from the observation that: “a lot of judges would say that <no> [to the thesis that the empathic judge can be at the same time impartial].” Then he examined the possibility that “empathy would be applied to all participants in the same way.” Dwelling on this, he mentioned the case in which “we have a crying lady, and the other does not cry, and we would say <I understand your situation>,” which in his opinion would undermine the impartiality. Another point relates to the problem with the civil cases between a private person and a company that has a legal standing—he asks, “how to be empathic toward the company.” Of course, real persons represent any collative body, but still, this argument shows that the symmetry thesis, as simple as it looks, blurs the fundamental differences between persons and entities involved in court trials.

Other judges also emphasized the weaknesses of the symmetry thesis from various perspectives. Some argued that impartiality does not equate to uniform behavior or demeanor [32], while others likened the attempt to feel equally toward both sides to a “schizophrenic endeavor” [23]. Additionally, another judge [31] noted that the behavior and reactions of other participants in the courtroom influence the space for the use of empathy by judges. In certain situations, judges may find it challenging to employ empathy due to factors such as a lack of cooperation from one party.

These critiques underscore the complexity of applying the symmetry thesis in practice and highlight the need for a more nuanced understanding of the role of empathy in judicial decision-making.

(2) One of the obvious strategies for defending the conformity (or at least the non-existence of non-conformity) between judges’ empathy and impartiality, also represented in the sample, is to accentuate the definition of empathy that does not suggest or imply a conflict between them (strategy of defining empathy as unemotional). Especially by emphasizing that empathy does not entail the dominance of emotions, but rather encompasses the understanding of someone’s situation or role. Conceptualizing empathy as carriers of emotionality poses serious problems for most judges, as emotions in general tend to be portrayed in professional culture as irritants in the judicial decision-making process (although this perception is gradually changing, even in Poland, due to the expansion of the law and emotion movement) [see in general: Maroney (2011); and in references to Polish judges: Wojciechowski et al. (2015)].

A typical representative of such a view is a statement by one of the judges [25], for whom “an empathic judge can be impartial, empathy that means understanding the position of the other side, but [at the same time] acknowledging a given legal context.” Similarly, another judge [34], in line with their understanding of empathy as being sensitive, noticed that “when the judge is sensitive to the needs of others and is not driven by emotions and does not tilt the scale to some party, [they] can be impartial. No one is a machine.” The slip into emotionality is dangerous, but empathy as sensitivity does not bring any problems.

Another example demonstrates something telling. A family judge [27] argued that “because empathy does not assume that we are sorely going to feel into, but that we going to understand the situation of one party, [and] only empathy allows for understanding the situation of both sides—thus, we are not losing the impartiality.” Later she admitted that “feeling into” is not possible in case of non-humans, but the “digging into the reasons of claims in the process” is possible. She clarified that empathy “it is not a case when one feels into the role of this person, this mother, this father, but empathy would allow us to maybe understand the situation of this person, why she acts this way, what her legal claim raised for, but not feeling into her role.” However, what is crucial is that her approach to empathy expressed at the beginning of the interview encompassed the feeling-into. This represents a shift in accents—to fit the argument, the judge changed the emotionally saturated understanding of empathy to one more reason-based process.

Certainly, in these instances, when addressing matters related to impartiality, there seems to be a scripted effort to detach emotionality from judging, refining empathy as not inherently linked with emotions. Consequently, the judges appear to gravitate toward equating empathy with “understanding the situation,” which is a safer option within the judicial context.

(3) Another “path” of merging empathy and impartiality explored by the judges involves strongly emphasizing that the role and impact of judges’ empathy do not concern the making of the final decision (strategy of mitigating empathy’s influence on judgments). In this way, the most important element of the process is presented as free from the problems and dilemmas that empathy—especially selective, partial, or strongly affective—brings. According to this line of thinking, empathy is pictured as a skill that works at the earlier phases of judicial decision-making. This strategy was most often mentioned by judges.

Exploring this avenue, a judge [35] elucidated: “I can understand both parties, but I will pass judgment which I think is just.” Along the reference to the symmetry thesis, the judge, in the last resort, will pass the decision not driven by empathy, but by other factors as well. Empathy does not destroy impartiality then as in the last resort, the final decision is not in any way influenced by what empathy gained. Similarly, one judge [30] realistically admitted that “the feeling informs [the proceedings], but rationality is what decides.” Furthermore, another interviewee [28] firmly stated that “the judgment is based on evidence and within the limits of the law—empathy helps but it is one element—empathy helps in questioning, contacts with people.” Echoing this sentiment, a judge [17] acknowledged that all people possess some kind of empathy and “I could be empathic to an old lady, but if her testimony does not suit the other material and seems not to be true, then I will not decide in her interest, yes. Thus, I will be impartial, I will try to be impartial, although I am not sure that I will be always successful.” The same approach was expressed by another judge [26] who stated that “empathy is needed for preparing everything for issuing the just, impartial judgment … but in the case of issuing the verdict, at this point, we are driven mostly by the binding laws.” This sentiment was echoed by several other judges [24, 10, 11].

(4) The next strategy employed by judges to reconcile the use of empathy with maintaining impartiality involves setting limits for empathy and highlighting certain associated dangers of its use (negative aspect). Additionally, judges undertake various forms of self-work, such as “distancing,” controlling, and self-monitoring, to mitigate these risks and ensure impartiality (positive aspect). In general, this strategy could be described as emphasizing control over empathy.

For example, a judge [8] claimed that there is no easy translation between being empathic and partial as “a judge must be aware of the different thought processes happening in his head and notice [them]. Extensive identification with one of the parties can lead to the balance being tilted in favor of that party. We are judges; we can consider, distinguish, and, as I am saying, [at the end] we are working by referring to the statutes.” Then, the judge referred to the comparison between judges and cooks, stating that beyond the ingredients, there is also a need for experiences, knowledge, and empathy. This metaphor stresses the role of the personal element in judging, which is unavoidable and desirable but should be under some self-monitoring by judges. The last stance refers to the earlier claim that empathy can work but before reaching judgment, here the judge assures that the decision is determined by the laws but not empathy, which stays under control. These two arguments nicely reinforce each other and together seem much stronger.

In a poignant reflection, a judge [16], while navigating the intricate relationship between empathy and impartiality, inadvertently intertwined empathy with compassion, lamenting: “there are situations in which the situation of a given person is so hard—simply my heart is broken, but I have to judge against her, on her disadvantage. Then, there is a huge boxing match with myself, but I cannot allow myself to be partial … this is an issue of ethos.” Here, the judge grapples with the emotional toll of difficult cases, acknowledging the internal struggle between empathic impulses and the imperative of impartial adjudication. Similarly, a judge [23, also 13], highlighted the importance of self-monitoring in preserving impartiality, stating: “but if it would happen during the trial, that I begin to tilt too much toward one party … then I immediately mitigate myself, that … [say to herself:] < wait a minute, I cannot go in this direction because I have to be basing on these facts, that I pass objectively make the decision>. Yes, I think, that adequately balancing these all ingredients can allow me to be an objective judge.” This introspective process of self-correction [done by self-talk—see Roach Anleu and Mack (2021: 105–106, 191–192)], a kind of internal work, serves as a crucial component of judicial ethos, ensuring that personal inclinations do not overshadow the objective assessment of facts and legal principles.

The judge from civil division [32], who possessed a deep understanding of empathy and its role in the judicial realm, provided profound insights into the impact of empathy-based biases and their management during trials. She articulated a conscientious approach toward recognizing and mitigating personal biases rooted in her life experiences, emphasizing the importance of maintaining impartiality. She expressed, “I try to be careful about my feelings which recall my life experiences and can understand such situations. And then I rather tend to think about myself, whether I favor [someone] and then I wonder whether I do not … lead toward the other side.” This statement underscores her self-awareness and vigilance in ensuring fair treatment of all parties involved. Furthermore, she described how her similar experiences with litigants prompt her to distance herself from identifying too closely with any party, activating “alert lamps” to signal potential biases. She elaborated about the internal mechanisms that occur during hearings, highlighting that while interactions and engagements create impulses, there is also a concurrent process of self-evaluation. This reflective capacity allows her, and other judges, to monitor their reactions and regulate their responses. She elucidated, “I can give time to myself, which gives me a chance to see the situation … it is not possible to be separated from one’s perspective, but it is possible to collect these moments in which I identify, in quotation marks, too hard, when someone’s perspective is close to me.” This demonstrates her ability to maintain perspective and discern when her empathetic identification with a party risk compromising impartiality, thereby enabling her to navigate empathy’s complexities in judicial decision-making.

Another judge outlined the process of managing impulses, illustrating how it contributes to achieving a state closer to objectivity. In an insightful anecdote, a judge [35] openly shared her struggle with a specific bias—her strong inclination to protect animal rights. She candidly acknowledged the challenge of navigating cases related to this subject matter, stating, “In such issues, one really needs to have the skills to close this inside oneself, to prevent them from surfacing, to treat people in a just way without imposing one’s worldview.” This admission not only demonstrates the judge’s self-awareness of potential biases but also underscores the delicate balancing act required to ensure a fair and unbiased decision-making process. The judge’s ability to recognize and control these inherent empathetic impulses is crucial. This self-awareness becomes a powerful tool in the pursuit of impartiality, with the judge actively working to mitigate the impact of personal biases on decision-making. However, the judge’s introspection also raises the important question of how she evaluates the adequacy of such control—whether it is a genuinely rationalized process or a potential area for further scrutiny and refinement. This highlights the ongoing challenge of achieving and maintaining impartiality in judicial decision-making, particularly when dealing with deeply ingrained personal convictions or biases.

(5) An interesting strategy for reconciling the use of empathy with impartiality is to avoid absolutizing or fetishizing impartiality in the face of real inequality between parties, or alternatively, to understand impartiality in a more justice-oriented manner rather than reading it as treating the parties identically. In the first scenario, impartiality is not necessarily the paramount value, creating room for empathy, while in the second, “just” impartiality may even necessitate empathy. This strategy can be called deabsolutizing formal impartiality and creating more space for empathy. Only two judges explored this avenue.

The first one [1], while acknowledging the crucial role of impartiality in the judicial process, astutely pointed out that impartiality should not be interpreted as ignoring the incompetence or ineptitude of any party involved. This perspective recognizes that the pursuit of justice cannot solely rely on strict adherence to formal impartiality when blatant imbalances or deficiencies exist. The judge further explored the complexities of maintaining balance between parties, suggesting that in cases where a lack of real balance is evident, actions may be justified that deviate from the rigid confines of formal impartiality. Similarly, the second judge [22] emphasized the importance of contextualizing impartiality within the broader framework of justice and fairness. While impartiality remains a fundamental principle of the judicial system, it cannot be divorced from the pursuit of equity and rectitude. The judge argued that rigidly adhering to impartiality without considering the unique circumstances and needs of each case risks perpetuating injustice rather than upholding true fairness. This insightful perspective reframes impartiality as a means to achieve justice rather than an end in itself, thereby allowing empathy to play a constructive role in promoting equitable outcomes.

## 8 Concluding discussion

The lack of acknowledgment of the potential incoherence between empathy and impartiality could be influenced by the socially desirable nature of empathy. Judges may strive to maintain both values or express a belief in their harmonious coexistence, especially given the importance placed on empathy in societal discourse. Additionally, the tense atmosphere surrounding the judiciary in Poland during the interviews could further influence judges to emphasize the significance of empathy in their decision-making processes, perhaps as a means to counter perceptions of judicial insensitivity spread by the then-ruling majority. In the face of such attacks, there could have been a mechanism of self-protection of the profession at play. However, it should be stressed that most of the judges did not fight to secure an ironclad picture of the judiciary but mentioned some of its problems and examples of unjust judicial behaviors.

Moreover, the total absence of voices suggesting that the two are conflicted can be surprising, considering the rather “positivistic” tradition and the continued dominance of the “stone face” ideal. However, as mentioned, Polish judges are hyper-active, and perhaps under the influence of other factors, they need to engage more empathic-like skills to navigate hearings, which could stimulate a greater openness to empathic engagement. However, the unanimous agreement among judges on the compatibility of empathy and impartiality could also suggest deeply ingrained beliefs within the judicial community. This alignment of perspectives indicates a prevailing consensus on the issue, and the research serves to elucidate and articulate these commonly held views rather than uncovering dissenting opinions. Testing these hypotheses would require a specific and dedicated research design.

Noticeably, the reconstructed “paths” of explaining the absence of contradiction between empathy and impartiality also invoke some problems and unanswered controversies. The symmetry thesis, often mentioned by judges, was criticized by some of the interviewees (see above). This is an easy way to make empathy and impartiality seem to be “friends,” but in fact, this strategy only pretends to solve the problem by arguing for a resolution that is in line with formal equality, which is easier for lawyers to accept. Moreover, it turns attention to the general claim of being equally empathic to each party, missing a plethora of problems and nuances. For example, Mack et al. (2021, 21) rightly mentioned that “empathy is not a zero-sum game; each party’s need for judicial empathy may be different.” Next, referring to the second strategy, some judges adjust their understanding of empathy to the circumstances—the topic of impartiality drove them to present views that are closer to “cognitive” empathy. Such instances of changing the accent during the interview could be interpreted as a way of securing the vision of professionalism of judicial officers and the activation of the script of judicial dispassion, activated without self-reflection. Moreover, acknowledging the lack of influence of empathy in passing judgment may, which forms the third strategy, seems sound, but it brings many issues—whether the judge indeed can control the internal processes in such a way and set such boundaries. Such a strategy can be interpreted as a kind of safe explanation. Moreover, the controlling theses that put emphasis on self-monitoring and self-disciplining seem promising but should be supplemented by more data based on the observation of judges and a more detailed description by judges of how these processes happen [how it looks in the case of Australian judges; see Mack et al. (2021: 9)]. In turn, the deabsolutizing of (formal) impartiality is certainly a bold argument. Although it was mentioned only by two interviewees, such a way of thinking leads to a rethink of the hierarchy of judicial values and principles.

Importantly, the judges were silent about the positive role of empathy in upholding judicial impartiality [see Lee (2013: 148)]. Only one judge [9] stressed that empathy “does not disturb impartiality, and even strengthens it. Absolutely, it is helpful.” However, this exception, although formulated as a general observation and without providing details, suggests that the possibility that empathy is, at least in certain situations and when properly deployed, an important antidote to biases, prejudices, and closed-mindedness was not on the participants’ radar. Even those judges who align with the understanding of empathy as general open-mindedness seem to not explore this avenue.

It is also crucial to note that employing in-depth interviews to grasp judges’ perceptions of the relationship between empathy and impartiality has inherent limitations. Such interviews primarily rely on verbal statements and may present an idealized picture, lacking self-critical examination, especially given the social roles of the interviewees. The study’s focus on verbal statements without comparing them with actual practices or observable actions may limit its depth. Interestingly, one judge [6], referring to himself as a “dinosaur” due to his extensive experience and adherence to traditional views, expressed concerns about the potential disparity between declarative statements and real-world application of empathy in judicial practice. He warned: “If we are going to talk about empathy, declaratively, the results will be quite good. However, in the real sphere, not necessarily. This is what I worry about, but today’s situation of courts and judges does not favor empathy and does not favor at all the understanding of the role of empathy; today [in 2021] we have a completely different direction [than encouraging more empathy].” This cautionary remark sheds light on the challenges of implementing empathy in the context of broader institutional dynamics and sociopolitical pressures toward judges. Additionally, it highlights the inclination to portray oneself in a more favorable manner.

## Data Availability

The datasets presented in this article are not readily available to protect the privacy of participants. Requests to access the datasets should be directed to mateusz.stepien@uj.edu.pl.

## References

[ref1] Bandes S. A. (2009). Empathetic judging and the rule of law. Cardozo Law Rev. De Novo, 133–148.

[ref2] Bandes S. A. (2011). Moral imagination in judging. Washburn Law Rev. 51, 1–24.

[ref3] Bednarek G. A. (2014). “Polish vs” in American courtroom discourse: Inquisitorial and adversarial procedures of witness examination in criminal trials (Basingstoke: Pengrave).

[ref4] Bergman Blix S. (2019). Different roads to empathy: stage actors and judges as polar cases. Emotions Soc. 1, 163–180. doi: 10.1332/263168919X15653390808962

[ref5] Bergman Blix S. Wettergren Å. (2018). Professional emotions in court. London: Routledge.

[ref6] Booth T. (2019). Family violence and judicial empathy: managing personal cross examination in Australian family law proceedings. Oñati Socio Legal Series 9, 702–725. doi: 10.35295/osls.iisl/0000-0000-0000-1037

[ref7] Braun V. Clarke V. (2006). Using thematic analysis in psychology. Qual. Res. Psychol. 3, 77–101. doi: 10.1191/1478088706qp063oa

[ref8] Colby T. B. (2012). In defense of judicial empathy. Minnesota Law Review 96, 1945–2015.

[ref9] Cuff B. Brown S. J. Taylor L. Howat D. J. (2016). Empathy: a review of the concept. Emot. Rev. 8, 144–153. doi: 10.1177/1754073914558466

[ref10] Čuroš P. (2023). Attack or reform: systemic interventions in the judiciary in Hungary, Poland, and Slovakia. Oñati Socio Legal Series 12, 626–658. doi: 10.35295/osls.iisl/0000-0000-0000-1393

[ref11] Dudek M. Stępień M. (2021). Courtroom power distance dynamics. Cham: Springer.

[ref12] Fissell B. (2017, 2016). Modern critiques of judicial empathy: a revised intellectual history. Michigan State Law Review 817, 817–851.

[ref13] Franks M. A. (2011). Lies, damned lies, and judicial empathy, Washburn L. J, vol. 51, 61–71.

[ref14] Glynn A. N. Sen M. (2015). Identifying judicial empathy: does having daughters cause judges to rule for Women’s issues? Am. J. Polit. Sci. 59, 37–54. doi: 10.1111/ajps.12118

[ref15] Gupta S. Harvey W. S. (2022). The highs and lows of interviewing legal elites. Int J Qual Methods 21, 160940692210787–160940692210711. doi: 10.1177/16094069221078733

[ref16] Guthridge M. Giummarra M. (2021). The taxonomy of empathy: a Meta-definition and the nine dimensions of the empathic system. J. Humanist. Psychol. 1:002216782110180. doi: 10.1177/00221678211018015

[ref9001] Henderson L. (1987). Legality and Empathy. Michigan Law Review 85, 1575–1577., PMID: 35663097

[ref17] Howlett M. (2022). Looking at the “field” through a zoom lens: methodological reflections on conducting online research during a global pandemic. Qual. Res. 22, 387–402. doi: 10.1177/1468794120985691, PMID: 35663097 PMC9095994

[ref18] Kenney S. J. (2020). Interviewing legal elites. London: SAGE Publications Limited.

[ref19] Korkea-aho E. Leino P. (2019). Interviewing lawyers: a critical self-reflection on expert interviews as a method of EU legal research. Eur. J. Legal Stud. 12, 17–47.

[ref20] Lanzoni S. (2018). Empathy: A history. New Haven, CT and London: Yale University Press.

[ref21] Lee R. K. (2013). Judging judges: empathy as the litmus test for impartiality. Univ. Cincinnati Law Rev. 82, 145–206.

[ref22] Mack K. Roach Anleu S. Tutton J. (2021). Judicial impartiality, Bias and emotion. Austr. J. Admin. Law 28, 66–82.

[ref9004] Maibom H. L. (2022). The space between. Oxford: Oxford University Press.

[ref9005] Maroney T. A. (2011). The Persistent Cultural Script of Judicial Dispassion. California Law Review 99, 629–681.

[ref23] Matthes C. Y. (2022). Judges as activists: how polish judges mobilise to defend the rule of law. East Eur. Politics 38, 468–487. doi: 10.1080/21599165.2022.2092843

[ref24] Merryman J. H. Pérez-Perdomo R. (2018). The civil law tradition: An introduction to the legal Systems of Europe and Latin America, fourth edition. 4th Edn. Redwood City: Stanford University Press.

[ref25] Mistygacz M. (2020). The position of the judge in Poland within the judicial system. Polit. Sci. Stud. 2020, 25–48. doi: 10.33896/SPolit.2020.58.2

[ref26] Pearson M. (2020). Empathy and procedural justice in clash of rights cases. Oxford J. Law Religion 9, 350–371. doi: 10.1093/ojlr/rwaa012

[ref27] Puleo L. Coman R. (2024). Explaining judges’ opposition when judicial independence is undermined: insights from Poland, Romania, and Hungary. Democratization 31, 47–69. doi: 10.1080/13510347.2023.2255833

[ref28] Roach Anleu S. Mack K. (2017). Performing judicial Authority in the Lower Courts. London: Palgrave.

[ref29] Roach Anleu S. Mack K. (2021). Judging and emotion a socio-legal analysis. London: Routledge.

[ref30] Rollert J. P. (2014). Standing in Barack Obama’s shoes: judging the President’s jurisprudence of empathy by James Wilson’s jurisprudence of common sense. Law Cult. Human. 10, 279–304. doi: 10.1177/1743872110393233

[ref31] Ryan A. (2016). Comparative procedural traditions: Poland’s journey from socialist to “adversarial’ system”. Int. J. Evid. Proof 20, 305–325. doi: 10.1177/1365712716655169

[ref9003] Sadurski (2019). Poland’s constitutional breakdown. Oxford: OUP.

[ref32] Salmons J. E. (2016). Doing qualitative research online. Thousand Oaks: Sage.

[ref34] Stępień M. (2021). On the relationship between judicial empathy and the integrity of judges. Krytyka Prawa. Niezależne Studia Nad Prawem 13, 98–113. doi: 10.7206/kp.2080-1084.474

[ref35] Szwed M. (2023). Fixing the problem of unlawfully appointed judges in Poland in the light of the ECHR. Hague J. Rule Law 15, 353–384. doi: 10.1007/s40803-023-00191-3

[ref9002] West R. (2011). The Anti-Empathic Turn. Georgetown Law Faculty Publications and Other Works. 678, 1–57.

[ref36] Wettergren Å. Bergman Blix S. (2016). Empathy and objectivity in the legal process: the case of Swedish prosecutors. J. Scandinavian Stud. Criminol. Crime Prevent. 17, 19–35. doi: 10.1080/14043858.2015.1136501

[ref37] Wojciechowski M. Dowgiałło B. Rancew-Sikora D. (2015). Emotional labour of judges. Archiwum Filozofii Prawa i Filozofii Społecznej 1, 97–109. doi: 10.36280/AFPiFS.2015.1.97

[ref38] Wood J. James M. Ciardha C. (2014). “I know how they must feel”: empathy and judging defendants. Eur. J. Psychol. Appl. Legal Context 6, 37–43. doi: 10.5093/ejpalc2014a5

[ref39] Zipursky B. C. (2012). “Anti-empathy and dispassionateness in adjudication” in Passions and emotions: NOMOS LIII. ed. Fleming J. E. (New York: NYU Press).

[ref40] Zoll F. Wortham L. (2019). Judicial Independence and accountability: withstanding political stress in Poland. Fordham Int. Law J. 42, 875–948.

